# FEELING CLOSER WITH FUTURE-SELF PROMOTES RETIREMENT PREPARATION OF YOUNGER AND OLDER WORKERS

**DOI:** 10.1093/geroni/igad104.3354

**Published:** 2023-12-21

**Authors:** Dannii Yeung, Esther Y K Tang, Gloria Lin, Alvin Ho

**Affiliations:** City University of Hong Kong, Hong Kong, Hong Kong; City University of Hong Kong, Hong Kong, Hong Kong; City University of Hong Kong, Hong Kong, Hong Kong; City University of Hong Kong, Hong Kong, Hong Kong

## Abstract

Working adults’ preparation for retirement and later life remains low despite past research has revealed its benefits on the well-being of retirees. This study utilized an experimental method to test whether the positive effect of exposure to future-self on retirement planning would be retained two weeks after the manipulation. Younger and middle-aged Hong Kong Chinese workers were recruited and were randomly assigned to one of the four conditions: exposure to a future-self photo plus a 5-day writing task (FSW), exposure to a future-self photo only (FS), exposure to a current-self photo plus a 5-day writing task (CSW), and exposure to a current-self photo only (CS). Participants in the FSW and CSW conditions were further asked to do a 5-day writing task. Intention to make retirement plans was assessed after viewing the future- or current-self photo (Time 1), while actual preparatory activities for retirement were also assessed two weeks after the photo viewing (Time 2). Preliminary analyses on 91 participants [41 younger (M=31.01,SD=5.55) and 51 middle-aged (M=50.04,SD=6.59) workers] showed significant interaction effects between condition and age on T2 overall retirement planning (F=4.06, p=.01, ηp2=.13) and financial planning (F=4.42, p=.006, ηp2=.14). Specifically, younger participants in the two future-self conditions had more preparatory activities than those in the CSW condition. Middle-aged workers in the FSW condition also reported more retirement preparation than those in the FS condition. These findings suggest that exposure to future-self increases retirement preparation in the short run, and such effect was stronger on younger workers than on older workers.

